# Headache prevalence and characteristics among school children as assessed by prospective paper diary recordings

**DOI:** 10.1007/s10194-011-0410-9

**Published:** 2011-12-27

**Authors:** Bo Larsson, Åsa Fichtel

**Affiliations:** 1 Department of Neuroscience, Faculty of Medicine, Regional Centre for Child and Adolescent Mental Health, Norwegian University of Science and Technology (NTNU), Trondheim, Norway; 2 Municipality of Uppsala, 73375 Uppsala, Sweden; 3 Department of Public Health and Caring Sciences, Uppsala University, Uppsala, Sweden

**Keywords:** Epidemiology, Assessment, Diary, Adolescents, School

## Abstract

In the present school-based study, a convenience sample of 477 students in grades 6–9 and second year in high school from a city and a smaller town recorded daily occurrence and intensity of headaches in a standard paper diary during a 3-week period. Total headache activity (headache sum), number of headache days, intensity level and duration for weekly headaches were estimated. Approximately 85% of the adolescents had experienced headache of any intensity level during the 3-week recording period. On the average, they reported 2.5 headache days per week and a mean intensity level for headache episodes of 1.7. Our estimates for headache of any intensity level (1–5) occurring at least once a week was surprisingly high (73.8%). For the highest intensity level across the whole 3-week period, almost identical proportions of mild and moderate headaches were reported by students (22.3–22.5%), while about twice as many (40.7%) had experienced severe headaches. Girls consistently reported more headaches than boys, in particular of the moderate and severe intensity types. Students in the city also reported more frequent and intense headaches than those in the town. Peak headache activity was observed at noon and in the afternoon and in the days from the middle of the week until weekend. The use of prospective recordings in diaries will further advance our knowledge on the prevalence and characteristics of recurrent headaches among children and adolescents in community samples.

## Introduction

Headaches are one of the most commonly reported health complaints in school children [1] and also the most frequently reported pain in these age groups [2]. Surveys conducted in Scandinavia and Holland indicate that school-aged children, in particular adolescent girls, report frequent headaches with an increasing prevalence during the last decade [3–6]. In a review of epidemiological studies of school-aged children, Lewis et al. [7] found the prevalence of any type of a significant headache to range from 37 to 51% in 7-year olds, gradually increasing to 57–82% by 15 years of age. Prevalence rates in school-aged children have been reported to be 23–51% for monthly headaches, 7–44% for weekly headaches, and 0.9–1.5% for daily or almost daily headaches [8].

While numerous studies since the 1950s have documented prevalence rates of unspecified headaches in children and adolescents, in most early studies, information was collected from health services based on physician or parent reports [9, 10]. However, given the low correspondence between parent and child reports of headaches in the child [11], during the recent decades, child and adolescent reports have been used much more often in epidemiological surveys. Although this is a definite progress in information quality, these reports have primarily been based on retrospect information in questionnaires covering various time intervals but also using different phrasings of headache items [12].

Given the variability of headache frequency, intensity and duration over time in children, adolescents and adults, prospective measurement is likely to reduce recall bias and produce more reliable and valid assessment than global single reports for the previous week or month-long period. As expected, the agreement is low between retrospective information sources such as questionnaires and interviews compared to prospective recording in diaries on estimates of headache and chronic pain prevalence among children in community populations [13–15]. In clinical studies of children primarily suffering from migraine [16–18], information obtained from questionnaires has been found to both overestimate and underestimate frequency and intensity of headache in children and adolescents as compared to prospective diary recordings.

To date, prevalence rates based on prospective recordings of headaches in a paper diary have been examined in unselected children aged 7–12 years and their parents in only one school-based study [11]. The authors found a 1-month prevalence rate of 57.6% (at least one headache episode reported) and that parents under-reported frequent headaches in the child compared to child report. Girls also reported more headaches than boys, in particular of the frequent type.

Given the paucity of surveys using prospective recordings of headaches in school-aged children in community populations by means of a diary, the aims of the present study were to estimate: (1) total headache activity (headache sum), number of headache days, headache intensity and duration when recorded during a 3-week period by children aged 11–18 years in a convenience school sample recruited from two locations (small town and city); (2) differences in regard to adolescent sex, age and school location; and (3) reliability of headache activity tested for associations over the 3-week period.

## Methods

### Sample

The present convenience sample of adolescents in the regular school population was recruited from one city (Uppsala with about 187,000 inhabitants, 2007) and a small town (Östhammar with about 4,500 inhabitants, 2007) in the same county in Sweden. In the city, the adolescents were recruited from five different schools consisting of four compulsory schools (grades 6–9) and one high school (11th year of schooling). In the town, one compulsory school participated (grades 6–9) and two classes from each grade were invited to participate in the study. The school principals at each school approved the study and selected the classes together with the teachers in the study. Information about the study was distributed by the classroom teacher to students and parents who were asked to inform the teacher if the child was not allowed to participate.

Out of a total of 550 invited school children, 8.7% (*n* = 48) were unable to participate due to their absence to school, or travelling, and demographic information on sex and age was incomplete for 25 students (5%). Thus, 477 adolescents were included in the study analysis and demographic characteristics of the sample are presented in Table 1.

**Table 1:** Demographic characteristics of the school sample by sex and grade

	Boys (*n* = 245)	Girls (*n* = 232)	Total (*N* = 477)
Grades			
6	61 (54.5%)	51 (45.5%)	112 (23.5%)
7	47 (48%)	51 (52%)	98 (20.5%)
8	58 (59.2%)	40 (40.8%)	98 (20.5%)
9	63 (52.1%)	58 (47.9%)	121 (25.4%)
11^a^	16 (33.3%)	32 (66.7%)	48 (10.1%)
Location			
City	147 (60%)	98 (40%)	245 (51.4%)
Town	132 (57%)	100 (43%)	232 (48.6%)

Number of subjects with percentages within parenthesis

^a^Second grade in high school

The students were asked to fill out a questionnaire on headache characteristics including ICHD2 criteria [19] and to keep a headache diary on paper for 3 weeks (see details below). Based on questionnaire information, the students with “headaches occurring last year” (*n* = 319; 65.5%) were classified as follows: migraine 10.4%; probable migraine (with one of the A-D criteria missing) 6.8%; tension-type headaches (TTH) 9.8%; probable TTH 16.1%; probable migraine or TTH 4.6%, and 15.9% unclassified headaches. For the first week, 90.8% of the students (*n* = 433; 49.9% boys) completed the diaries, for the second week 85.5% (*n* = 408; 51% boys), and for the third week 80.7% (*n* = 385; 51.4% boys). Three complete weeks of diary recordings were performed by 71.7% (*n* = 342) of the students, while 2 weeks of recordings were obtained from another 15.1% (*n* = 72) of the enrolled sample, 8.0% (*n* = 38) recorded headaches for 1 week, and 5.2% (*n* = 25) did not produce any diary recordings.

### Headache diary

The paper diary developed by Budzynski and collaborators [20] and revised by Epstein and Abel [21] was chosen because of its extensive use in the previous controlled outcome studies on adults with recurrent headaches [22] as well as in our own controlled trials of the effects of school-based treatments for adolescents aged 10–18 years [23] and by others [18]. The diary has been socially validated against ratings by significant others in adults [22] treated because of recurrent headaches as well as for adolescents [24]. For children with recurrent migraine attacks and adults with frequent migraine or tension-type headaches, a 3-week recording period has been found to be optimal in regard to compliance, validity and reliability of ratings [25].

The adolescents were asked to record their headaches four times a day at approximately 5 h intervals (at breakfast: 7 a.m.; at lunch: 12 a.m.; in the afternoon: 17 p.m., and at bedtime: 22 p.m.) on a 0–5 intensity scale (see Table 2).

**Table 2:** Headache diary rating scale

0 = No headache
1 = Very mild headache, only noticeable when attending to it
2 = Mild headache, could be ignored at times
3 = Moderate headache, normal activities can be continued
4 = Severe headache, difficult to concentrate, can manage undemanding tasks
5 = Extremely intense headache, incapacitated, cannot do anything

From the weekly diary, the following measures were extracted: total weekly headache activity, *headache sum* = sum of all the 28 weekly ratings with a score range of 0–140; number of *headache days* = count days when any headache activity was recorded; *Headache intensity* = mean of ratings of headache episodes (score range 1–5). In line with guidelines developed by the IHS [26], the raw intensity scale (0–5) was also recoded to a 0–3 scale (0 = No headache; 1–2 = mild; 3 = moderate; 4–5 = severe headache). *Headache duration* = mean length of headache episodes based on the number of consecutively reported time points of any headache activity in a given day (range 0–4).

Research assistants visited each classroom weekly to collect the diaries and also reminded teachers and participants to continue the headache recordings for the following week. The study was conducted in the middle of the spring semester and each class was paid 1,500 Swedish crowns (about 200 US dollars) after data collection was terminated.

The study conformed to the revised ethical principles of the Helsinki declaration.

### Statistics

Descriptive statistics included means, standard deviations and percentages. Chi-square test was used to examine associations between categorical variables, while Pearson product–moment correlations were used for continuous variables. Differences between independent group means were examined by Student *t* test or ANOVA. For repeated measurements, ANOVA was used including testing of possible linear or quadratic effects across the 3-week period. When significant main effects were found, subsequent post hoc testing included Bonferroni contrasts. Effect sizes were calculated using Cohen’s partial η^2^ [27] and interpreted according to the criteria for the percent variance accounted for: small effect it is 1–5.9%, medium effect 6.0–13.8%, and above 13.8% indicates a large effect. Missing values in the diaries were imputed for each individual if less than 15% of their measurement points across the 3-week recordings were omitted using the expectation maximization (EM) procedure [28]. A *p* value of 0.05 or less indicated statistical significance for two-tailed tests and SPSS 17.0 was used in the analyses.

## Results

Mean values and SDs for the headache sum, headache days, intensity and duration for weekly episodes by sex, age and location are presented in Table 3.

**Table 3:** Means (SDs within parenthesis) for weekly total headache activity (headache sum), number of headache days, intensity and duration for the 3-week diary recording period by student sex, grade, school location and assessment point

	Headache sum	Headache days	Intensity^b^	Duration^b,c^
	(0–140)	(0–7)	(1–5)	(1–4)
All	8.5 (10.1)	2.5 (2.1)	1.7 (0.7)	1.6 (0.6)
Sex				
Boys	6.8 (8.7)	2.0 (1.9)	1.7 (0.7)	1.6 (0.7)
Girls	10.3 (11.2)	3.0 (2.1)	1.8 (0.7)	1.6 (0.6)
Grades				
6	6.9 (7.8)	2.2 (1.9)	1.7 (0.6)	1.5 (0.6)
7	9.9 (12.2)	2.5 (2.2)	1.9 (0.8)	1.7 (0.6)
8	7.4 (9.0)	2.4 (2.2)	1.8 (0.8)	1.6 (0.7)
9	9.1 (11.8)	2.6 (2.0)	1.6 (0.5)	1.7 (0.7)
11^a^	9.5 (7.5)	3.2 (1.8)	1.6 (0.4)	1.7 (0.5)
School location				
City	8.8 (9.8)	2.8 (2.1)	1.6 (0.5)	1.6 (0.6)
Town	8.0 (10.6)	2.0 (1.9)	1.9 (0.8)	1.7 (0.7)
Time point				
Week one	8.7 (12.2)	2.6 (2.4)	1.6 (0.6)	1.6 (0.7)
Week two	8.2 (11.6)	2.4 (2.3)	1.7 (0.7)	1.6 (0.8)
Week three	8.4 (12.2)	2.4 (2.4)	1.7 (0.8)	1.7 (0.8)

^a^Second grade in high school

^b^Intensity and duration scores calculated for headache episodes

^c^Unit length 5 h

### Headache sum

The mean score for total headache activity across the 3-week period was 8.5 (SD 10.2). The 90th percentile for headache sum scores ranged 20–22 across the 3-week period. The results of a 3-way ANOVA (sex by grade by location) showed a significant effect for sex, (*p* = 0.001) (ES 2.6%) in that girls reported significantly more total headache activity than boys. The results of a 3-way ANOVA with repeated measurement showed a significant time by location interaction linear effect, (*p* = 0.016) (ES 1.8%), but not for any other interactions or main effects. The results of post hoc test showed that students in the city increased their total headache activity scores between the weeks 2 and 3, whereas those in the town decreased theirs significantly (*p* = 0.005).

**Fig. 1:**
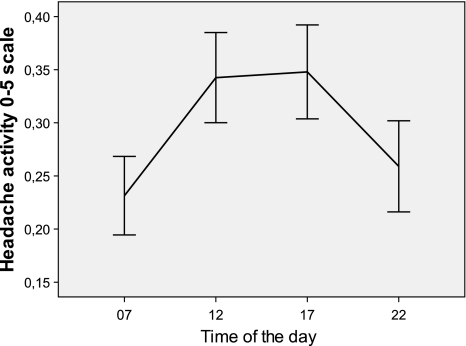
Mean headache intensity as rated on a 0–5 scale at four daily time points

**Fig. 2:**
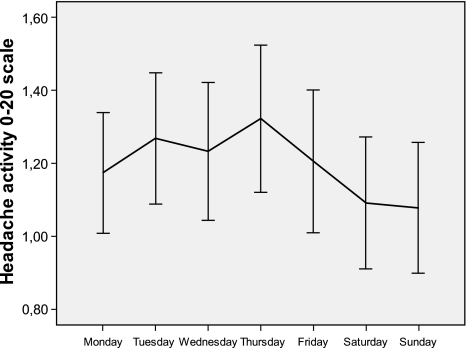
Mean daily headache activity across all weekdays as rated on a 0–20 scale

Across the four daily reporting points, there was a peak for both boys and girls at noon and afternoon for average headache activity with girls showing significantly higher scores than boys, (*p* = 0.012) (ES 1.9%) (see Fig. 1). A significant sex by time quadratic effect was also found, (*p* = 0.022) (ES 1.6%). When analysing changes in daily headache activity across the seven weekdays, a significant weekday by grade linear interaction effect was obtained, (*p* = 0.035) (ES 3.1%); however, none of the post hoc contrasts was significant (see Fig. 2).

### Headache days per week

For the whole 3-week period, 14.8% of the students did not report any headache, 11.3% had headaches 1–3 times, 43% had headaches 1–3.5 times a week, and 30.8% had daily or almost daily headaches.

On the average, the adolescents reported 2.5 headache days per week (having an intensity score of 1 or more; SD 2.1). The results of a 3-way ANOVA showed significant effects for sex, (*p* < 0.001) (ES 5.5%) and location, (*p* < 0.001) (ES 4.6%). Girls reported significantly more headache days than boys, and students in the city had significantly higher number of headache days than those in the town. A significant grade by location interaction effect was also obtained, (*p* < 0.001) (ES 4.2%). Subsequent post hoc analysis showed that students in the city and grade 7 reported significantly (*p* = 0.003) more headache days compared to those in the 6th grade, while the reverse was found for those in the town.

The results of 3-way ANOVA with repeated measurement showed a significant time effect, (*p* = 0.017) (ES 1.7%), indicating that number of headache days decreased significantly across the 3 weeks. A significant linear time by location interaction effect was also obtained, (*p* = 0.001) (ES 3.2%), with headache days increasing slightly for students in the city over time, while a pronounced decrease was found for those in the town. Finally, a significant grade by location interaction effect was obtained, (*p* = 0.007) (ES 3.7%), with students in the city and in grade 7 reporting significantly (*p* = 0.003) greater number of weekly headache days than those in grade 6 which contrasted to adolescents in the town and grade 7 where the reverse was found.

### Headache intensity

For the whole sample, mean intensity level for headache episodes (scale 1–5) across the 3-week period was 1.7 (SD 0.65). The results of a 3-way ANOVA showed a significant effect for grade, (*p* = 0.021) (ES 3.1%) and location (*p* = 0.001) (ES 3.2%). Subsequent post hoc test revealed that students in grade 7 reported significantly higher intensity levels than those in grade 9 (*p* = 0.007) and 11 (*p* = 0.021). No significant interaction effect was found.

Further analysis for headache intensity and duration was also carried out by means of a 2-way ANOVA (sex by grade effect) with repeated measurement for students (*n* = 172) with complete headache episode data across the 3-week recording period. The results showed a significant linear effect for time, (*p* = 0.006) (ES 4.5%) in that adolescents reported significantly (*p* = 0.006) higher intensity scores during week three as compared to week one.

Almost identical proportions of mild and moderate headaches were reported by adolescents (22.3–22.5%), while about twice as many (40.7%) had experienced severe headaches (see Table 4). Moderate and severe headaches were found to be significantly (*p* = 0.005) more common among girls than boys. However, the relationships with grade and location were nonsignificant.

**Table 4:** Percentage of students with no headache, mild, moderate and severe headaches as the highest intensity score recorded across the 3-week diary period by sex, grade, and school location

	No headache	Mild^b^	Moderate^c^	Severe^d^
	(*n* = 61)	(*n* = 93)	(*n* = 94)	(*n* = 171)
All	14.5	22.5	22.3	40.7
Sex				
Boys	19.7	25.0	19.7	35.6
Girls	9.3	20.0	24.9	45.9
Grades				
6	15.3	21.6	21.6	41.4
7	13.9	20.8	18.1	47.2
8	17.9	24.4	26.9	30.8
9	14.5	27.3	18.2	40.0
11^a^	8.3	10.4	33.3	47.9
School location				
City	12.1	23.0	25.4	39.5
Town	18.1	21.1	18.1	42.7

^a^Second grade in high school

^b^Raw intensity score of 1–2

^c^Raw intensity score of 3

^d^Raw intensity score of 4–5

Over the 3-week period about half of the adolescents (55.2%) reported varying weekly headache intensity levels (a score of 0–5), and one quarter (24.9%) had experienced moderate headaches across the whole period or at least 2 weeks with severe headaches, while 5.3% had had severe headache episodes during each of the 3 weeks.

### Headache duration

For the whole sample, the overall unit mean length of headache episodes was 1.6 (SD 0.62). No difference in average duration scores was obtained for the whole 3-week period by sex, grade or location. While the results of a 2-way ANOVA with repeated measurement revealed a significant main effect for time, (*p* = 0.044) (ES 2.5%), subsequent post hoc tests were nonsignificant.

### Relationships between number of headache days and intensity

The majority of students with less than one headache day per week reported mild headaches, and those with 1–3.5 headache days per week reported equal levels of mild and moderate intensity levels, while almost half of them had experienced severe headaches (45.3%). About two-thirds (67%) of the students who reported daily or almost daily headaches did also experience them as being of moderate or severe intensity.

### Correlations between weeks 1–3

The relationships between the three weekly headache sum scores were *r* = 0.53 for week one versus week two, *r* = 0.49 for week one versus week three, and *r* = 0.62 for week two versus week three (all *p* < 0.001). While about one quarter of students with headaches 1–3.5 times during week one, were free of headaches one or two weeks later, about two-thirds of the students with daily or almost daily headaches (3.5 days or more) continued to report such headaches over the first 2 weeks, while the corresponding figure for week one–week three was 57%.

## Discussion

In the present study of a convenience school sample, 477 students in grades 6–9 and second year in high school recorded the occurrence and intensity of headaches in a standard paper diary format during a 3-week period. The overall results showed that a substantial number of students (85%) had experienced headaches of any intensity level (a score of 1–5) during this limited recording period, and 40% had had at least one episode of severe headache.

In contrast to the present study and a previous Norwegian school-based survey [11] both being based on prospective diary recordings of current headaches, numerous surveys have investigated headache prevalence among adolescents, most commonly assessed retrospectively in 6 or 12 month or lifetime perspectives (see Grimmer et al. [12]). To date, very few epidemiological surveys have included information on current headaches or headaches experienced in a short-term perspective. Fendrich and colleagues [29] reported that 31.6% of 12- to 15-year-old German adolescents had headaches during the previous week when information was elicited on retrospect questionnaire data. An almost identical figure (31.4%) was reported for headaches occurring during the last week among 10- to 18-year-old school adolescents in a Brazilian study by Barea and colleagues [30] who used interviews. A higher figure, 47%, was noted for headaches occurring during the last week in a Dutch epidemiological study of school children [6]. In a nationwide Swedish study [31], twice as many 9- to 15-year-old girls (17%) as boys (8%) reported headaches at least once a week during the last 10–14 weeks as evaluated with a questionnaire. Overall, the prevalence estimates in these surveys contrast to the much higher figures obtained in the present study in which about three quarters of adolescents prospectively reported unspecified frequent headaches of any intensity level occurring at least once a week.

Almost all the previous epidemiological research on the prevalence and characteristics of headaches among children and adolescents has been based on retrospective recall with varying time intervals and questionnaires with different phrasings of headache items. To date, only one previous study has been conducted in which children in an unselected school sample and their parents prospectively recorded headaches in the child during a 6-week period [11]. Somewhat more than half of the children (57.6%) aged 7–12 recorded a headache episode across the whole period with an almost identical figure for parents. The authors concluded that their 1-month prevalence figure was higher than previously reported in questionnaire-based surveys for the same age group. In their study, 42.4% had no headache, 42.9% had headaches about 1–3 times a month, 10.4% had about 1–3 headache days per week, and 4.3% of the children had daily or almost daily headaches during the 6-week period.

In the present study, in which adolescents recorded any headaches on a 5-point scale, only a small portion, 14.8%, did not report any headache at all (a score of 1 or more) over the 3-week period, while 11.3% had headaches 1–3 times, 43% had headaches 1–3.5 times a week, and 30.8% had daily or almost daily headaches. These strikingly higher estimates of headache frequency than previously reported in surveys of schoolchildren (typically 7–44%) [8] are very likely due to the use of prospective diary recordings and the inclusion of very mild headaches (defined as “only noticeable when attending to it”) which commonly occur at these ages. Quite consistent with these findings, in a previous school-based study [14], one-third of children who reported no headaches in questionnaires or interviews reported at least one headache episode in a diary, however, the majority of these were of low intensity.

The highest reported intensity score across the 3-week period was also subgrouped into mild, moderate and severe categories as recommended by the IHS trials subcommittee in evaluations of treatment outcome [26]. While such a categorization of headaches is clinically and socially relevant, it has yet been used in only a few epidemiological surveys of children and adolescents. In a study of adolescents using questionnaires and interviews, approximately the same proportions of students reported mild or moderate headache intensity (43.1 and 46.6%, respectively), and considerably fewer (10.3%) had severe headaches [32].

A finding in the present study was that intensity of headaches was positively related to frequency, i.e., students with daily or almost daily headaches also reported them to be severe, while those with low-frequent headaches experienced them as being mild. Similar associations have also been reported among school children with chronic pain syndromes [33].

In the present study, peak headache activity among school adolescents was observed at noon and later in the afternoon, and in the middle of the week until the weekend. This finding is likely to be related to adolescents’ experience of school stress which accumulates through the day as well as the week also emphasized in cross-sectional studies of relationships between recurrent headaches and various psychosocial stressors [34, 35].

To date, findings on agreement between diary, questionnaire and interview-based information on childhood headache are inconsistent. In a previous study of school children with migraine and tension-type headaches, estimates of headache frequency were much higher in diary recordings, in particular of low-intensity levels compared to questionnaires and interviews [14]. In a study of children and adolescents with recurrent headaches, van den Brink and colleagues [13] observed that headache frequency was both underestimated and overestimated in questionnaires when compared to a 4-week diary recording period, while intensity and duration levels of headaches were overestimated. Overall, the correlations between questionnaires and diary for headache intensity, frequency and duration were low. Metsähonkala and collaborators [15] noted that attack duration was significantly longer as recorded in the dairy than interviews but frequency of attacks was equal in a study of 11- to 13-year-old children with migraine who recorded their attacks shortly after each episode for a 2- to 7-month period.

In the present study, the correlations between weekly measurements of total headache activity as recorded in the diary were moderate (*r* = 0.49 − 0.62) and lower than estimates (*r* = 0.85 − 0.99) reported for recurrent migraine among school adolescents recruited to a treatment study [25]. While weekly headache intensity levels varied across the 3-week period, stability of daily or almost daily headaches among adolescents in the present study was high for moderate or severe intensity levels.

Some limitations of the present study need to be noted. It included a convenience sample and teachers selected the participating classes. Headaches among students were also assessed for a limited 3-week period in the middle of spring when school work load and stress is generally high which might have produced overestimates of headache occurrence. Because headaches fluctuate over time, the representativeness of this measurement period is uncertain. Although students were asked to record their headache complaints four times a day, it is unknown to what degree they adhered to the predetermined times or relied on recall. The completion rate also declined somewhat over the 3-week period possibly due to student tiredness in the recordings which is likely to reduce the reliability and validity of the headache data. The use of a handheld electronic diary has been shown to be feasible, and to produce greater compliance and accuracy than paper diary in prospective recordings of headaches and other pain in children and adolescents in small group clinical studies [36, 37]. However, its validity, reliability and psychometric properties still need to be tested in comparisons to paper formats [38] being more practical in large-scale epidemiological surveys of headaches in children and adolescents.

## Conclusion

The strengths of the study are the relatively large sample including adolescents in an unselected school population from schools in a city and a smaller town in the same county also serving rural areas and age groups in which recurrent headaches of different intensity levels are common. The overall response rate was also high. To our knowledge, this is the first epidemiological study in which school adolescents prospectively recorded their headaches in a daily diary. Our estimates of headaches occurring at least once a week, or daily or almost daily among the adolescents were surprisingly high as well as the proportion of severe headaches in the present unselected sample of adolescents who reported on their headaches in a short-term perspective. The striking differences in estimates when compared to numerous epidemiological surveys conducted primarily by means of questionnaires and retrospective information are likely to depend on the use of prospective diary recordings performed by the students during a 3-week period and the inclusion of low-intensity headaches. Such a method is important to use in the assessment of frequent headaches in children and adolescents in clinical practice as well as school health service because it captures all headache intensity levels in these age groups.
